# An American Text-Book of the Diseases of Children

**Published:** 1899-01

**Authors:** 


AN AMERICAN TEXT-BOOK OF THE DISEASES OF CHILDREN. Edited by LOUIS STARR, M. D., Consulting Pediatrist to the Maternity Hospital, Philadelphia. Second edition, revised and enlarged. Handsome imperial octavo volume of 1,244 pages, profusely illustrated. Prices, cloth, $7.00 net; sheep or half morocco, $8.00 net. Philadelphia: W. B. Saunders. 1898.

When an American text-book of the diseases of children made its appearance, four years ago, it was a work whose merits were so evident that it found its way into almost every physician's library. It was one of the first, if not the very first, of the modern American books on pediatrics. Since that time there have appeared a number of treatises by eminent authorities on diseases of children, yet even with the variety of works from which to choose, we feel that he will make no mistake who in buying for a small medical library selects an American text-book for his authority on pediatrics.

Like all the works in the American text-book series, it is bulky and unweildly, yet the publisher doubtless has his own reasons for presenting these works in an inconvenient form.

The first edition has been thoroughly revised, some articles rewritten, and others added. Among the additions is a brief, though useful, article by Dr. Thompson S. Westcott, on Modified milk and percentage milk mixtures, by reference to which the physician beyond the reach of milk laboratories will be able to wrestle successfully with the intricacies of percentage feeding. Dr. Rachford, of Cincinnati, contributes a new and valuable chapter on lithemia, a condition which not unfrequently passes unrecognised in infancy and childhood. This chapter is a very readable one, and will doubtless help the physician to understand many an obscure case.

Those familiar with Dr. Rochford's work recognise his right to speak with authority. Dr. James E. Moore, of the University of Minnesota, contributes a chapter on orthopedics, a subject not included in the old edition.

The chapters on typhoid fever, rubella, chicken-pox, tuberculosis, meningitis, hydrocephalus and scurvy have been entirely rewritten; those on infant feeding, measles, diphtheria and cretinism more or less completely revised, the volume being thus increased in size by fully fifty pages.

To sum up the matter, there is presented in this revised edition of Starr's text-book of the diseases of children an up-to-date treatise, whose merits are so well known and appreciated that to point them out would be superfluous. Indeed, the early appearance of a second edition is itself the best commentary on the rank and value of the work. M. J. F.

